# The role of casein in supporting the operation of surface bound kinesin

**DOI:** 10.1186/1754-1611-2-14

**Published:** 2008-10-20

**Authors:** Vivek Verma, William O Hancock, Jeffrey M Catchmark

**Affiliations:** 1 Engineering Science and Mechanics, University Park, PA 16802, USA; 2 Department of Bioengineering, University Park, PA 16802, USA; 3 Agricultural and Biological Engineering, University Park, PA 16802, USA

## Abstract

Microtubules and associated motor proteins such as kinesin are envisioned for applications such as bioseparation and molecular sorting to powering hybrid synthetic mechanical devices. One of the challenges in realizing such systems is retaining motor functionality on device surfaces. Kinesin motors adsorbed onto glass surfaces lose their functionality or ability to interact with microtubules if not adsorbed with other supporting proteins. Casein, a milk protein, is commonly used in microtubule motility assays to preserve kinesin functionality. However, the mechanism responsible for this preservation of motor function is unknown. To study casein and kinesin interaction, a series of microtubule motility assays were performed where whole milk casein, or its α_s1 _and α_s2_, β or κ subunits, were introduced or omitted at various steps of the motility assay. In addition, a series of epifluorescence and total internal reflection microscopy (TIRF) experiments were conducted where fluorescently labeled casein was introduced at various steps of the motility assay to assess casein-casein and casein-glass binding dynamics. From these experiments it is concluded that casein forms a bi-layer which supports the operation of kinesin. The first tightly bound layer of casein mainly performs the function of anchoring the kinesin while the second more loosely bound layer of casein positions the head domain of the kinesin to more optimally interact with microtubules. Studies on individual casein subunits indicate that β casein was most effective in supporting kinesin functionality while κ casein was found to be least effective.

## Background

Biological molecular motors are a unique class of proteins, which exist in eukaryotic cells and function as nano scale vehicles that drive a range of fundamental biological processes. Kinesin motor proteins transport intracellular cargo, move proteins and mRNA in neurons, and play a vital role in cell division. These motor proteins move unidirectionally on protein tracks known as microtubules which assemble and disassemble creating a unique dynamically reconfigurable transportation system. Kinesins are powered by the hydrolysis of adenosine triphosphate (ATP) and exert a maximal force of ~6 pN and exhibit a maximal efficiency of ~50% [1-3]. Biological motors are the subject of intense research in part because of their potential to be used *in-vitro *as 'nano-engines' for several future applications ranging from bioseparation to powering hybrid micro and nano scale electromechanical systems (MEMS/NEMS) [4-8]. Surface interactions and adsorption of molecular motors have also been studied [9,10].

An important hurdle that must be overcome before realizing these applications is developing approaches for preserving motor protein function when integrated into engineered devices and surfaces. Motor protein function is studied using gliding assays or bead assays [11,12]. In the bead assay, microtubules are immobilized on a surface and biological motors attached to microscale beads move along these immobilized microtubules. In the gliding assay, the motor proteins are immobilized on a surface and microtubules are transported across the surface by the immobilized motors. In either case, these motor proteins are attached to a surface in a manner that is intended to preserve their functionality. This is typically accomplished via the use of blocker proteins that prevent motor denaturation on the untreated surface [11,13]. We studied microtubule motility through gliding assay.

The most common proteins used for creating kinesin compatible surface interfaces are caseins extracted from bovine milk [12-14]. These proteins have been extensively used in the surface immobilization of motor proteins such as kinesin, and they are also used in Western Blots for blocking nonspecific adsorption of antibodies to nitrocellulose membranes. A typical process for performing a gliding assay consists of: 1) constructing a flow cell using a glass slide (Fisher Finest Premium microscope slides) and glass cover slip (Corning 1 1/2, 18 mm^2^); 2) blocking the glass surfaces by flowing in a solution of 0.5 mg/ml casein protein; 3) adsorbing kinesin to the glass surface by flowing in a solution of kinesin containing ATP and 0.2 mg/ml casein; 4) flowing in a microtubule solution containing ATP, antifade reagents and casein, and 5) observing microtubule movements by epi-fluorescence microscopy. Microtubule motility is quantified by examining several parameters including the density of microtubules observed, their landing rate on the surface, the microtubule transport velocity, and the distances microtubules move before detaching from the surface and diffusing away.

The initial blocking step is implemented to form a layer of the protein casein on the surface to prevent the kinesin denaturation that would occur if the motors proteins were directly adsorbed to the glass surface [11]. Casein is included in the motor and microtubule solutions based on a qualitative observation that it improves microtubule motility. However, although casein is used to optimize motor activity in a number of different in vitro motility assays on surfaces, no studies have been conducted to understand the mechanism by which casein creates compatible interfaces. Understanding the role of casein in supporting biomotor function is important for developing design rules for engineering optimal surfaces for use in advanced hybrid devices that incorporate biological motor proteins for actuation and transport.

Caseins are phosphoproteins that bind calcium and form large aggregates in milk. Casein subunits range from 20–30 kDa and are classified into 4 types: α_s1_, α_s2_, β, and κ. The relative concentrations of these caseins vary depends upon the milk producing species. Casein from bovine milk, which is commonly used in motor protein experiments, contains the following subunit compositions in skim milk (mg/ml): α_s1 _(12%–15%), α_s2 _(3%–4%), β (9%–11%), and κ (2%–4%) [15,16]. The different forms of casein have been extensively studied due to their importance in milk, as food additives and as emulsifiers and stabilizers for glue, paint, and other materials [17]. Although there are no crystal structures of any of the subunits, every one of the subunits contains one or more clearly defined hydrophobic and hydrophilic domains, resulting in the formation of micelles with differing geometries [18-21].

**Figure 1:**
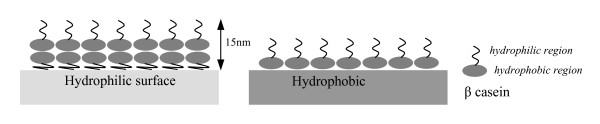
**Schematic illustration of the adsorption of β casein on hydrophilic and hydrophobic surfaces**. In the case of a hydrophilic surface, a bi-layer structure forms with a loosely bound second layer which measures ~15 nm thick [22].

The dynamics of casein adsorption onto surfaces has been studied using various approaches. Specifically, adsorption of β casein onto hydrophilic and hydrophobic surfaces has been studied by Nylander, et al. [22] and Kull, et. al [23]. The β casein protein has one distinct hydrophilic and hydrophobic domain, which gives the molecule a strong amphiphilic character [15]. It has been found that on hydrophobic surfaces β casein forms a monolayer where its hydrophobic region is positioned next to the hydrophobic surface and its hydrophilic region is protruding outward into the aqueous solution. However, in the case of a hydrophilic surface, the hydrophilic domain of the β casein protein adsorbs into the surface resulting in a tightly packed monolayer producing a hydrophobic surface. A second more loosely bound layer of β casein is then adsorbed onto the first layer where its hydrophobic region is aligned with the hydrophobic region of the first adsorbed casein layer and its hydrophilic region is positioned outward into the aqueous solution. The thickness of the bi-layer has been measured to be ~15 nm [22]. This is illustrated in figure 1.

In the present work, we have examined the role of both whole casein (consisting of mixture of α_s1_, α_s2_, β, and κ casein) and the various casein subunits in modulating kinesin function in the microtubule gliding assay. In particular, microtubule motility assays were performed where casein was either included or excluded in the surface blocking, kinesin adsorption, and microtubule solutions. The inclusion of casein in various incubation steps and the specific casein subunits used had dramatic impacts on the functionality of surface adsorbed kinesin, as measured by counting the number of microtubules on the surface. Most notably, samples including κ casein exhibited significant reductions in the number of microtubules observed. Studies on fluorescently labeled casein were also performed where the labeled casein was introduced into the flow-cell surface either as a first or after unlabeled casein had been adsorbed. By measuring the binding of fluorescent casein to the surface, we hypothesize that casein forms a bi-layer on the glass surface and that optimum kinesin-microtubule binding is achieved when kinesin proteins co-assemble with the casein bi-layer.

## Methods

Casein was prepared by dissolving the protein powder (Sigma C-7078, St Louis, MO) in BRB80 buffer (80 mM PIPES, 1 mM MgCl_2_, 1 mM EGTA, pH 6.9), centrifuged to remove un-dissolved casein. The solution was then filtered using a 220 nm filter (Fisher brand, Ireland). Full length *Drosophila *conventional kinesin heavy chain and lacking light chains was bacterially expressed and purified according to established procedures [24]. Tubulin was prepared from bovine brain and rhodamine-labeled according to standard procedures [25,26]. Microtubules were polymerized by incubating a mixture of 32 μM tubulin, 4 mM MgCl_2_, 1 mM GTP, and 5% DMSO in BRB80 buffer at 37°C for 20 minutes, and were stabilized by diluting to a final concentration of 0.32 μM in a solution containing 10 μM paclitaxel. Kinesin was labeled with Rhodamine-NHS using the standard protocol for tubulin labeling [25] to examine relative density of kinesin absorbing to the surface in the presence of different casein.

All the motility experiments were performed in a closed volume flow cell as previously described [13,27]. Flow cells consisted of a microscope slide (Fisher Finest Premium microscope slides) and a glass cover slip (Corning 1 1/2, 18 mm^2^) separated by double side tape, and had volumes of ~20 μl. The protein solutions were exchanged in these flow cells and motility of microtubules was assessed. In a flow cell, solutions were exchanged by injecting fresh buffer from one end and wicking out the previous solution from the other. In control experiments the first step is surface blocking where 0.5 mg/ml casein is incubated in the flow cell for 10 minutes followed by a solution of kinesin motors (0.05–5 μg/ml) including 0.2 mg/ml casein and 100 μM Mg-ATP in BRB80 buffer. Finally, microtubule motility solution containing 0.032 μM microtubules, 10 μM paclitaxel, 0.2 mg/ml casein, 1 mM Mg-ATP, 20 mM D-glucose, 20 μg/ml glucose oxidase, 8 μg/ml catalase and 0.5% β-merceptoethanol in BRB80 buffer is introduced. Microtubules were observed by epifluorescence microscopy (Nikon E600, 100×, 1.3 N. A. oil immersion Plan Fluor objective) and captured to videotape using a Genwac GW-902H camera. Microtubule surface binding was quantified by counting the number of microtubules present in a given video screen (65 μm × 48 μm) after 15 minute incubation time. Microtubules with length longer than 1 μm and moving for more than 2 μm were counted. Immobilized microtubules or those smaller than 1 μm were not counted. In all cases microtubule binding refers to microtubule binding to and moved by kinesin.

Epifluorescence and total internal reflection microscopy (TIRF) using a Nikon TE2000 inverted microscope (60×, 1.45 NA, CFI Plan Apo TIRF oil objective) was used to measure fluorescent intensities to study bi-layer formation of casein, and co-assembly between kinesin and casein proteins. Meta-Vue (Universal Imaging, PA) software was used to acquire images for bi-layer and co-assembly studies using rhodamine casein and rhodamine kinesin. Fluorescent intensities from the surface from the recorded images were measured in arbitrary units.

## Results and discussion

To assess the role that casein is playing in supporting the activity of surface-adsorbed kinesin, we first examined kinesin function under a range of casein conditions and then quantified the binding of both casein and kinesin motors to the surfaces using fluorescence assays. While surface-adsorbed motors are difficult to visualize microscopically, it is relatively straightforward to visualize and quantify the interaction of microtubules with these motors. It has been shown previously that because individual kinesin motors are sufficient to bind microtubules and transport them across the surface, below maximal motor densities, the rate that microtubules land and move over the surface is proportional to the density of active kinesins on the surface [11,24]. Hence, the surface density of functional motors can be estimated by allowing microtubules to land for a defined time and then counting the number of microtubules on the surface. We systematically removed casein from either the blocking solution, the motor solution, or the microtubule solution and counted the average number of microtubules propagating over a kinesin functionalized glass surface after 15 minutes. Whole bovine milk casein, containing all four casein subunits, was used, and both high (8 μg/ml) and low (0.8 μg/ml) concentrations of kinesin protein were examined. It has been shown previously that at low kinesin concentrations, very few or no motors are functionally adsorbed to the surface if the surfaces are not pretreated with a blocking protein, but at high motor concentrations some fraction of the motors presumably bind and denature on the surface and replace the role of blocking proteins [11]. However, to date, the role of casein in the motor and microtubule solutions has not been systematically investigated.

Table 1 shows the measured number of microtubules propagating in a 65 μm × 48 μm video screen area under various casein loading conditions using either high or low kinesin concentrations. The numbers of microtubules were counted from five screen shots and their mean and standard deviation was calculated. From this data three clear observations can be made. First, if there is no casein blocking step and no casein is included in the motor solution then no microtubules are observed. This result, which is consistent with previous work [11] suggests that in the absence of a casein treatment to block the surface, the kinesin motors denature on the glass surface or bind such that their motor domains cannot interact with microtubules. Second, the initial casein blocking step does not significantly impact the microtubule motility when casein is included in the subsequent kinesin adsorption solution. The inclusion of a casein blocking step improved the number of observed microtubules from 60 ± 4.4 (Mean ± standard deviation, N = 5) to 80 ± 9.6 (33% improvement) for high kinesin concentrations, and from 4.4 ± 1.5 to 9.2 ± 2.4 (109% improvement) for low kinesin concentrations. Presumably, because the casein concentration (200 μg/ml) is considerably higher than the motor concentration (8 or 0.8 μg/ml), the casein binds rapidly to the surface and is able to carry out its blocking role if it is included in the motor solution. Third, the inclusion of casein in the microtubule solution always increases the observed number of microtubules, with an average improvement of 3-fold over all conditions, and a maximum observed improvement of 5.4-fold in the case where casein is included in both the blocking and kinesin adsorption steps and a high kinesin concentration was used.

**Table 1:** Measured average number of microtubules observed under different casein and kinesin concentration conditions.

Casein Blocking	Kinesin concentration	Casein in Kinesin	Casein in MT	Average MT per screen (N = 5)
No	Low (0.8 μg/ml)	No	No	0 ± 0
			
			Yes	0 ± 0
		
		Yes	No	1.4 ± 1.3
			
			Yes	4.4 ± 1.5
	
	High (8 μg/ml)	No	No	0 ± 0
			
			Yes	0 ± 0
		
		Yes	No	9.4 ± 1.1
			
			Yes	60 ± 4.4

Yes	Low (0.8 μg/ml)	No	No	0 ± 0
			
			Yes	0 ± 0
		
		Yes	No	3.6 ± 1.5
			
			Yes	9.2 ± 2.4
	
	High (8 μg/ml)	No	No	13.6 ± 2.4
			
			Yes	48 ± 5.7
		
		Yes	No	20 ± 3
			
			Yes	80 ± 9.6

N: Number of screen shots.

The observed variation in the number of microtubules present on the surface under differing experimental conditions must then result from the impact of casein on either the availability of surface adsorbed kinesin to participate in microtubule binding, or the density of kinesin adsorbed. From table 1 it can be concluded that surface adsorbed kinesin retain their functionality except in the case of low kinesin concentration and where no casein has been included in any incubation solution. The loss of kinesin functionality can either be due to denaturing of the kinesin or binding of the kinesin head domain to the surface, which may result in no observed microtubule binding. Hence, the inclusion of casein is not impacting the ability of the kinesin to operate normally on an available microtubule if contact with a microtubule was possible. This hypothesis is based on the observation of increased observed microtubule density when casein is added to the final microtubule solution. Moreover, since no additional kinesin had been added in the microtubule solution with casein, the density of kinesin on the surface could not have been increased, but rather more of the existing kinesin on the surface were available to participate in microtubule binding when casein was included in the microtubule solution. It is also apparent that once kinesin are adsorbed in the absence of casein, they cannot be revived by subsequent inclusion of casein in the microtubule solution. This may result from kinesin having a stronger affinity for the surface than casein or that, in the case of kinesin denaturing on the glass surface, that denatured kinesin cannot regain their functionality if casein is subsequently introduced and is able to displace the kinesin.

**Figure 2:**
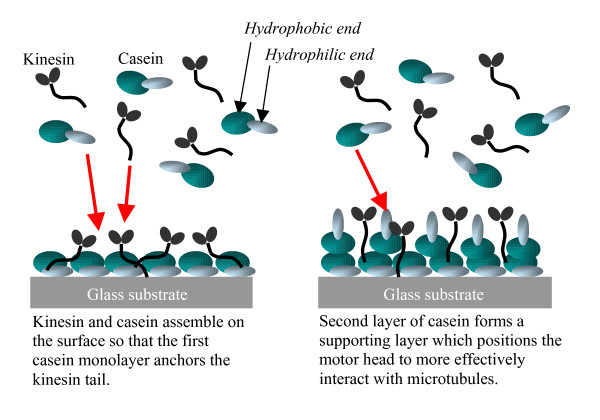
**Schematic illustration of the assembly process of casein and kinesin**. Kinesin are bound to the surface through interaction with the first adsorbed layer of casein. The second more loosely bound layer of casein interacts with the head domain of the kinesin to promote interaction with microtubules.

Based on these observations, we hypothesize that casein is forming a bi-layer on the surface of the glass substrate in a manner similar to that previously reported [22]. In our model, the kinesin assemble onto the first strongly adsorbed layer of casein which anchor the kinesin to the surface. Although the mechanism of this interaction needs to be identified, it is possible that the kinesin tail has an affinity for some region on the casein protein or that the kinesin tail has an affinity for the glass surface but the casein protein prevents the kinesin head from binding to the glass surface. In any case, it is clear that the kinesin have a stronger affinity to the first layer of casein than the second layer of casein has for the first layer of casein or the kinesin. The second more loosely bound layer of casein serves to position the head region of the kinesin promoting interaction with a microtubule. The observed increase in microtubule binding when casein was included in the microtubule solution suggests that the kinesin are fixed with some affinity to their position since the solution exchange does not remove the kinesin from the surface to a significant extent (see below). However, when casein was included in the microtubule solution the number of microtubules observed increases in all cases suggesting that an increase in the interaction of adsorbed motors with microtubules occurs in the presence of additional casein, which replenishes the second loosely bound casein layer which is disrupted during the final microtubule solution exchange. This co-assembly model is shown in figure 2.

In the model illustrated in figure 2, the casein is assumed to have one distinct hydrophilic domain and one distinct hydrophobic domain, as is true for β-casein [22]. However, the casein used for the experiments summarized in table 1 included casein from bovine milk which consists of α_s1_(12%–15%), α_s2_(3%–4%), β (9%–11%), and κ (2%–4%) caseins. The structure of these caseins have been determined [21] and are not identical. One of the most pronounced differences is in the structure of the two α caseins, which have two distinct hydrophobic domains.

**Figure 3:**
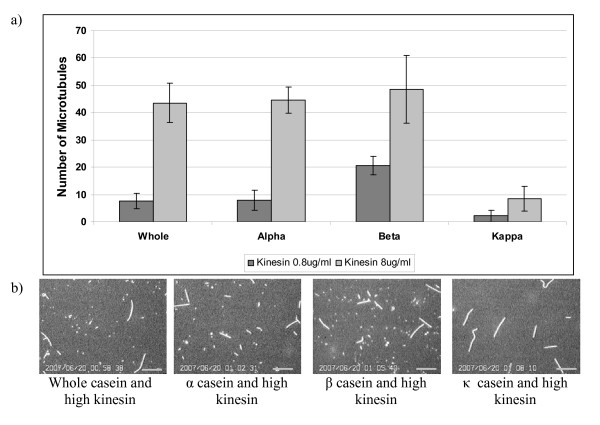
**Comparing the effectiveness of different casein subunits on kinesin function**. (a) The average number of microtubules was observed in a standard motility assay at low (0.8 μg/ml) and high (8 μg/ml) motor concentrations where casein was included in the surface blocking, kinesin adsorption and microtubule motility steps. For different casein subunits, (α, β and κ) all solutions contained only that specific subunit. (b) Screen shots showing microtubule density on glass surface for different casein and high kinesin. Microtubules less than 1 μm were not counted. Scale bar is 10 μm.

To determine if the individual casein subunits impact the kinesin-microtubule interaction differently, we performed motility assays using purified forms of α (containing both α_s1 _and α_s2 _subunits)_,β _and κ caseins, and compared these results to a control using whole bovine milk casein. The average number of microtubules observed under each condition is shown in figure 3(a). In figure 3(b) screen shots of microtubule motility assay for high kinesin concentration are included. The results for assays containing purified form of α casein matched results from control assays using whole casein. This finding is consistent with the fact that bovine milk contains 15%–19% α casein, while β caseins and κ caseins are present in smaller proportions of 9%–11% and 2%–4%, respectively. Interestingly, β casein was most effective in supporting microtubule motility, leading to a 1.6-fold increase compared to whole casein or α casein at low motor densities. This may suggest that the decrease in the average number of microtubules observed for whole and α casein vs. β casein may in fact result from the different caseins interacting to form heterogeneous bi-layer structures where either of the two casein layers contain two or more adsorbed casein subunits (see below). Finally, κ caseins appear to be the least effective in supporting kinesin-microtubule interaction. In κ casein there were only 20% and 29% as many microtubules as in whole casein at high and low motor densities, respectively. The finding that κ casein leads to low activity even for high motor concentrations suggests that kinesin interacts differently with κ casein than with α or β casein.

The differences observed in the function of the different casein subunits presumably derive from the different structures of the various casein subunits. Because the α caseins have two hydrophobic domains, the packing and order of its bi-layer should quantitatively differ from the structure of the bi-layer from β casein, which contains one distinct hydrophobic domain in addition to its hydrophilic domain. Moreover, κ casein contains only one hydrophilic and hydrophobic domain like β casein, but the average number of microtubules observed for assays containing κ casein was nearly an order of magnitude lower than that observed for assays containing β casein (figure 3). However, κ casein is thought to play a role in casein micelle assembly [19-21] that is distinct from the role of α and β subunits. Interestingly, in virtually all microtubule motility studies no binding of microtubule whose length less than 2–3 μm was observed in case of κ casein.

**Figure 4:**
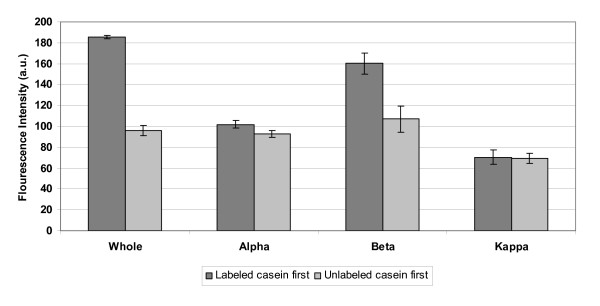
**Determining if various caseins form bi-layer where first layer is bound strongly to the glass**. In first set rhodamine labeled casein was incubated in flow cell, washed with antifade solution and the fluorescent intensity was measured using total internal reflection microscopy (TIRF). In the second set, unlabeled casein was incubated before injecting in the labeled casein. The flowcell was washed with antifade solution and fluorescence was measured using TIRF.

To determine whether all of the casein subunits are assembling into a bi-layer on their own in a similar manner that whole casein is presumed to assemble, the binding of fluorescent casein subunits to a glass surface was measured by fluorescence microscopy. Glass coverslips were coated with rhodamine labeled whole, α, β and κ casein, washed in an antifade solution containing BRB80, 20 mM D-glucose, 20 μg/ml glucose oxidase, 8 μg/ml catalase and 0.5% β-merceptoethanol to reduce rhodamine bleaching, and visualized using epi-fluorescence microscopy. A second set of glass cover slips were first coated with unlabeled casein, then exposed to fluorescently-labeled casein, and finally antifade solution was washed in. The intensity of the rhodamine fluorescence on the surface was quantified for each case and the intensity corrected for the background signal in the absence of any fluorescent label. Data for the two conditions are presented in figure 4. Rhodamine labeling extent of casein and its subunits was taken into consideration. UV spectrophotometer analysis was performed on rhodamine labeled caseins and the fluorescence intensity was normalized accordingly. When rhodamine labeled whole casein or casein subunits was exposed to the clean glass surface, a tightly bound layer of casein adsorbed to the surface that was not washed away by replacing the protein solution with the antifade solution. However, when unlabeled casein was first introduced into the flow cell followed by the labeled casein, the measured intensity decreased in all cases. In the case of whole casein and β casein, this decrease was substantial, representing a reduction of 48% and 34%, respectively. These data suggest that a bi-layer structure is formed for the whole casein and β casein, and that the second layer is more loosely bound. The very small difference in the measured intensities for κ casein again suggests a difference in how this casein interacts with the glass surface and kinesin and provides no evidence for formation of a bi-layer. For α casein there was less overall binding and the pretreatment with unlabeled casein had a minimal effect. The microtubule binding data from figure 3 suggest that whole casein and α casein interact similarly with surface-adsorbed kinesin, but the binding data in figure 4 imply the opposite and instead suggest similarities between whole casein and β casein. It is possible that the difference rests in the specific interactions between the different α_s1 _and α_s2 _caseins, which are not examined in isolation here. Alternatively, it is possible that in the case of the whole casein, the individual caseins form a heterogeneous bi-layer that integrates different casein subunits. For example, the first adsorbed layer could be an α casein whose hydrophobic region interacts with the hydrophobic region of a β casein, etc. Importantly, the maximum observed number of microtubules in figure 3 was observed for pure β casein, and both figure 4 and previous work suggest that β casein forms a bi-layer on the surface [22], suggesting that a casein bi-layer is an important component in promoting effective kinesin-microtubule interaction.

**Figure 5:**
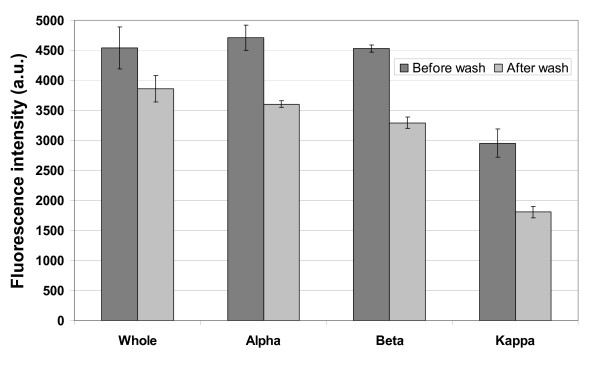
**Fluorescent intensity (arbitrary units) of rhodamine labeled kinesin as measured using total internal reflection microscopy**. The flow cell was incubated for 10 minutes with whole casein or different casein subunit solutions. Rhodamine labeled kinesin was then injected into the flow cell and the fluorescence was measured using total internal reflection microscopy (TIRF). Finally, the flow cells were injected with antifade solution and fluorescence was again measured by TIRF.

While measuring microtubule binding is a good readout for the density of active kinesin motors on the surface, it cannot differentiate between changes in the concentration of motors adsorbed to the surface and the relative activity of the adsorbed motors. To examine the relative density of kinesin that adsorbs to the surface in the presence of the different casein subunits and to determine the impact of solution exchange on surface-adsorbed kinesin surface density, the binding of fluorescent kinesin motors to the surface was measured by fluorescence microscopy. In this experiment a glass slide was first blocked with unlabeled casein and then a solution containing rhodamine labeled kinesin and unlabeled casein was injected into the flow cell and incubated for 30 minutes. The density of surface-bound motors was estimated by measuring the fluorescence intensity at the surface by total internal reflection fluorescence (TIRF) microscopy as described in methods. Because this technique only measures fluorescence within ~100 nm of the surface, the contribution from any unbound motors in the solution is minimized. Finally this motor solution was replaced with a solution containing casein with no additional kinesin. This solution was incubated for 30 minutes and the surface fluorescence was again quantified using TIRF. Figure 5 shows the measured fluorescence intensity values after background subtraction before and after the wash step. The first result is that before washout the density of motors was similar for whole, α and β casein, while fewer motors bound in the presence of κ casein. The second result is that the washout decreased the kinesin surface density by 15%, 24%, 27% and 39% for whole, α, β and κ caseins, respectively. The fact that the density of kinesin is only partially reduced by the solution exchange step indicates that the kinesin are either bound to the surface or to the first layer of casein adsorbed to the surface. One notable result is that both before and after washout the fluorescence intensity was the lowest for κ casein, suggesting that κ casein is the least effective at enabling kinesin to bind to the surface. This result is consistent with the small number of microtubules observed for κ casein in figure 3, but the ~55% reduction in the density of adsorbed motors does not fully account nearly order of magnitude decrease in the number of microtubules bound in κ casein or that κ casein can not support kinesin functionality. Collectively, this data may suggest that κ casein reduces the interaction between kinesin and microtubule relative to the other casein subunits. This may result from the inability of κ casein to form a bi-layer as shown in figure 4. More research is needed to elucidate the differences in the interactions between the individual caseins and kinesin.

## Conclusion

The impact of the presence of both typical bovine milk casein and purified α_s1 _and α_s2 _(combined)_,β _and κ caseins on the binding of microtubules on casein-kinesin coated glass surfaces has been examined. Microtubule motility assays were performed where casein was either included or excluded in the surface blocking, kinesin adsorption and microtubule solutions. It is found that the inclusion of casein at the various incubation steps has a dramatic impact on the number of microtubules observed binding to kinesin on the surface. No microtubules are observed on the surface when casein is not included in the kinesin adsorption solution and the inclusion of casein in the microtubule solution always increases the observed number of microtubules. Moreover, there were quantitative differences in the number of microtubules observed when purified forms of the different caseins were used. Most notably, samples including κ casein exhibited significant reductions in the number of microtubules observed. Studies on fluorescently labeled casein were also performed where the labeled casein was introduced to the surface either first or after an unlabeled casein had been adsorbed. Fluorescence intensities were notably higher in the case where the labeled casein was introduced to the surface first, except in the case of α and κ casein.

From this data a bi-layer casein adsorption model is proposed where the first tightly bound layer of casein mainly performs the function of anchoring the kinesin while the second more loosely bound layer of casein positions the head domain of the kinesin to more optimally interact with microtubules. Studies on adsorption of fluorescently labeled caseins in the first and second layers support the bi-layer model of casein assembly. Results using whole casein from bovine milk are also compared to that obtained when using purified α_s1 _and α_s2 _(combined)_,β _and κ caseins and significant variations in observed number of microtubules are found. β casein is found to be the best at promoting kinesin-microtubule interaction while κ casein is found to be the worst, and may behave completely different than the other caseins including the lack of a bi-layer assembly.

Kinesin and microtubule system is studied extensively to design hybrid synthetic devices for detection and transport. Motility assays are also used in studying the role of kinesin, *in vitro*, which may help get an insight on how to control cell division. Some of the assays, however, require more specific engineered surfaces than others and it is important to understand the mechanism which supports operation of surface bound kinesin. In this paper, we propose a bi-layer model based on our studies. However, more studies are needed to understand the interaction of kinesin with the various caseins. A more detailed understanding may provide new insights enabling the design of engineered surfaces for optimally supporting kinesin activity, a key parameter in the development of hybrid biological-synthetic devices incorporating biological molecular motors.

## Competing interests

The authors declare that they have no competing interests.

## Authors' contributions

VV carried out all the experiments. JMC, VV and WOH contributed to the manuscript preparation. VV and JMC conceived the studies and designed the experiments. JMC, VV and WOH contributed to the discussion and bi-layer model. All authors have read and approved the final manuscript.
